# Supplementary material for: Origin and Evolution of the Cannabinoid Oxidocyclase Gene Family
Source: Genome Biol Evol. 2021 Jun 8;13(8):evab130. doi: 10.1093/gbe/evab130 (PMC8521752; doi:10.1093/gbe/evab130)
Supplement: evab130_Supplementary_Data [file evab130_supplementary_data.zip › Supplementary figure legends_210102.docx]

# Supplementary figure legends

Fig. S1. Cannabinoid oxidocyclase gene tree based on nucleotide sequences from genbank accessions. Clade B was used as an outgroup. Labels indicate genbank accession; putative nonfunctional (pseudo)genes are in grey; functionally characterized THCAS and CBDAS [(Sirikantaramas et al. 2004; Taura, Sirikantaramas, Shoyama, Yoshikai, et al. 2007)](https://paperpile.com/c/GuJfGV/NBmW+dgDd) are indicated in dark red. Coloured blocks indicate the identified clades; white blocks indicate sequence types. Node labels indicate posterior probabilities below 1.0.

Fig. S2. Cannabinoid oxidocyclase gene tree based on nucleotide sequences from whole-genome assemblies of cultivars ‘Cannatonic’, ‘Chemdog91’, ‘Jamaican Lion’ (father), ‘LA confidential’, and ‘Pineapple Banana Bubble Kush’ (PBBK). Clade B was used as outgroup. Labels indicate genbank accession of genomic contig and locus tag (when available) or start position. Putative nonfunctional (pseudo)genes are in grey; functionally characterized THCAS, CBDAS, and CBCAS [(Sirikantaramas et al. 2004; Taura, Sirikantaramas, Shoyama, Yoshikai, et al. 2007; Laverty et al. 2019)](https://paperpile.com/c/GuJfGV/pt6c+NBmW+dgDd) are indicated in dark red. Coloured blocks indicate the identified clades; white blocks indicate sequence types. Node labels indicate posterior probabilities below 1.0.

Fig. S3. LASTZ nucleotide alignment dotplots of microsyntenic cluster 1 showing clade C tandemly repeated array and (A) CBDAS, or (B) THCAS variants. Sense alignments are in black; antisense alignments are in blue. Triangles indicate start positions of genes. For gene color codes see Fig. 4A.

Fig. S4. LASTZ nucleotide alignment dotplots of microsyntenic cluster 2 showing CBCAS tandemly repeated array. Sense alignments are in black; antisense alignments are in blue. Triangles indicate start positions of genes. For gene color codes see Fig. 4B.
