# Supplementary figures and images for: Origin and Evolution of the Cannabinoid Oxidocyclase Gene Family
Source: Genome Biol Evol. 2021 Jun 8;13(8):evab130. doi: 10.1093/gbe/evab130 (PMC8521752; doi:10.1093/gbe/evab130)

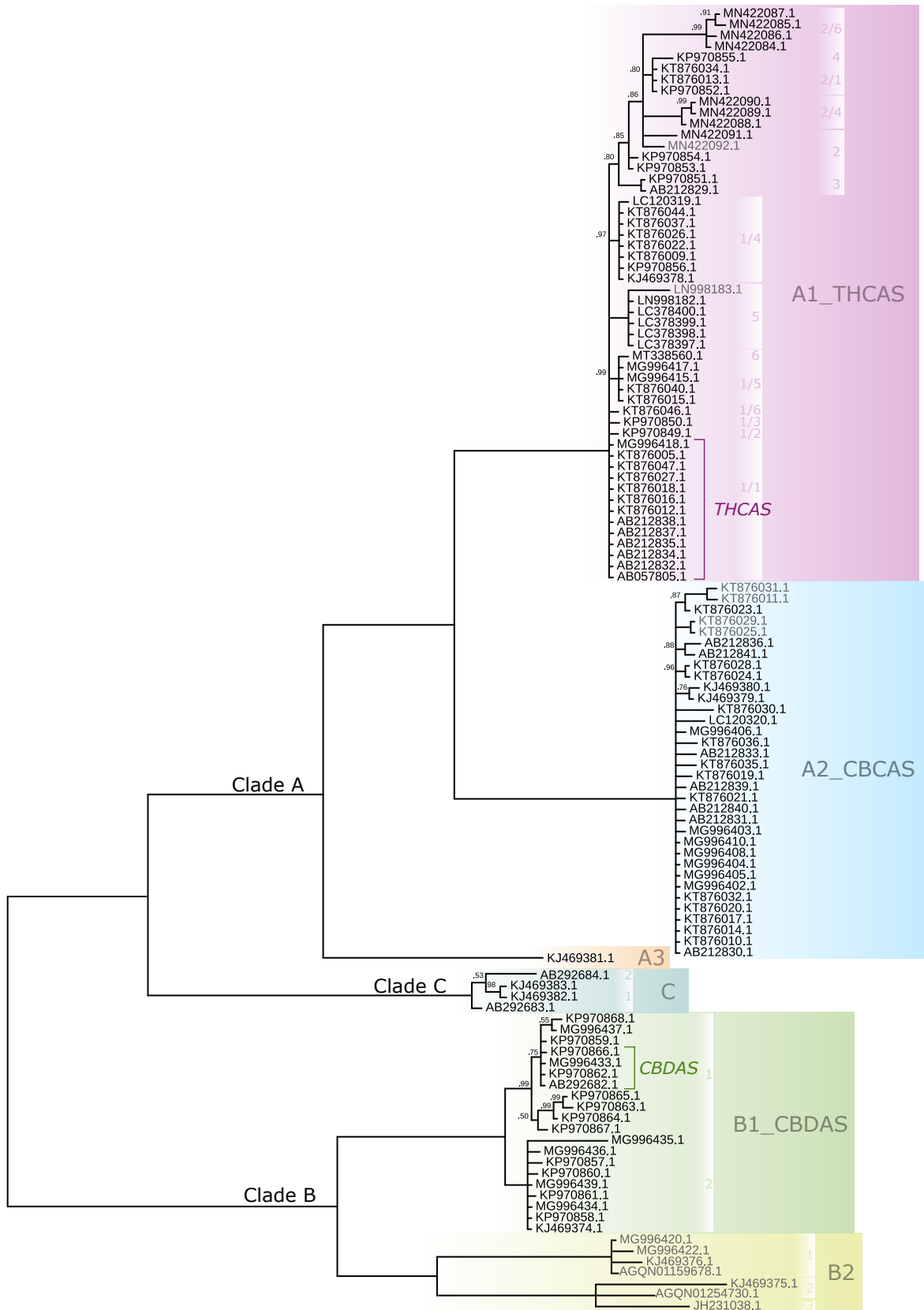

Supplement: evab130_Supplementary_Data [file evab130_supplementary_data.zip › Figure S1 - Cannabinoid oxidocyclase gene tree based on genbank accessions_v2.pdf]

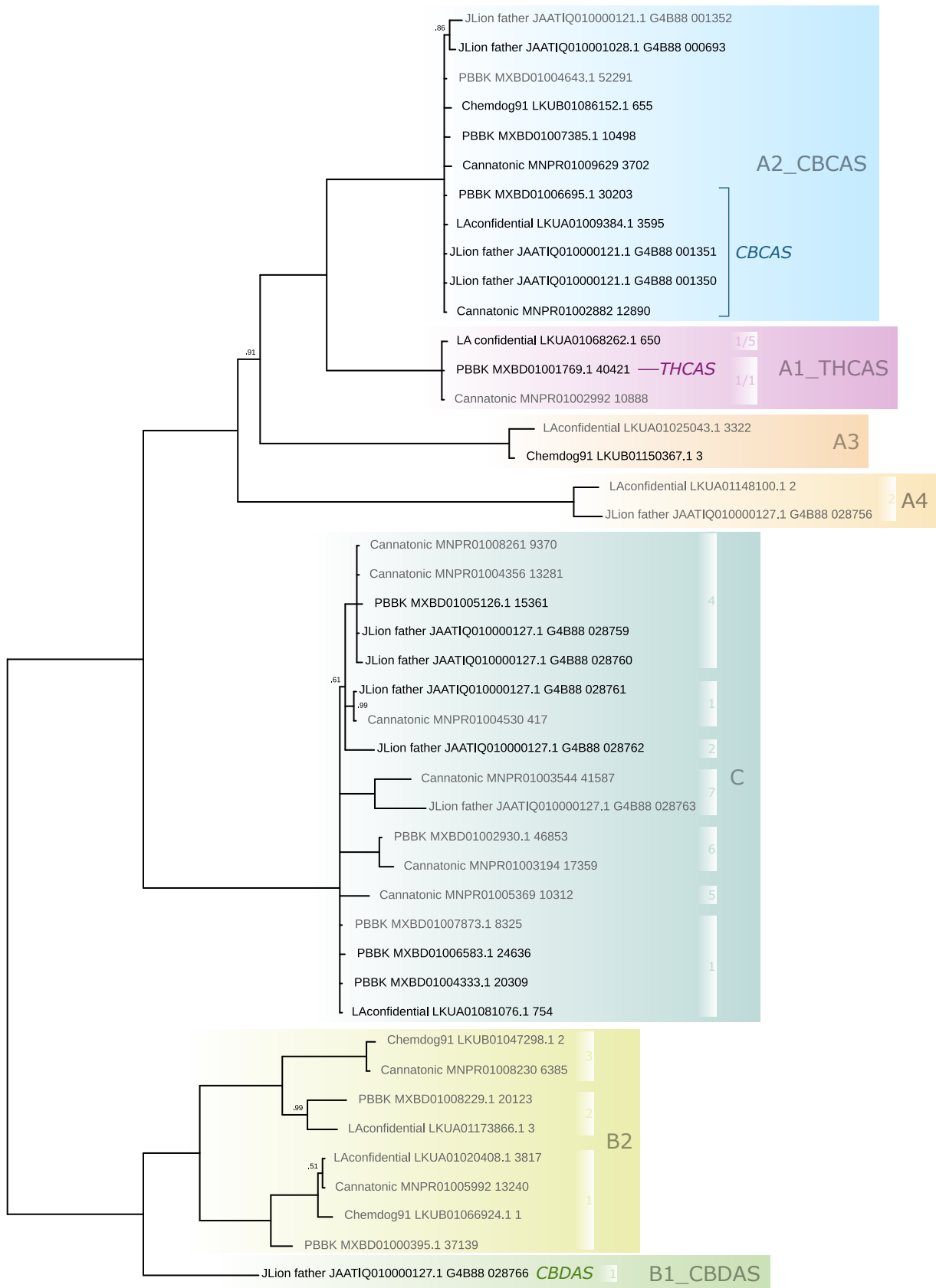

Supplement: evab130_Supplementary_Data [file evab130_supplementary_data.zip › Figure S2 - Cannabinoid oxidocyclase gene tree based on whole-genome assemblies_v2.pdf]

CBDRx

Jamaican Lion (mother)

CBDRx

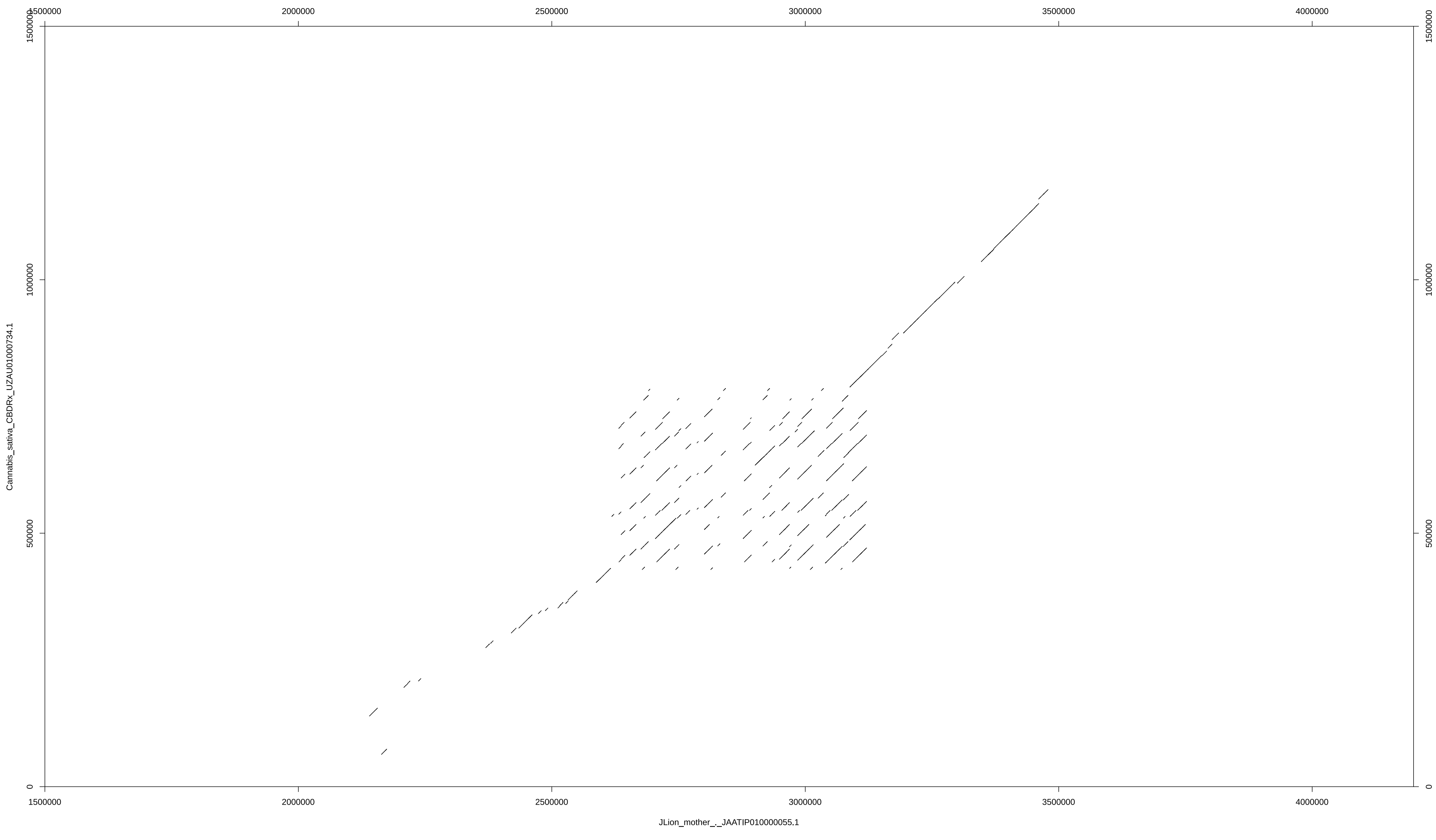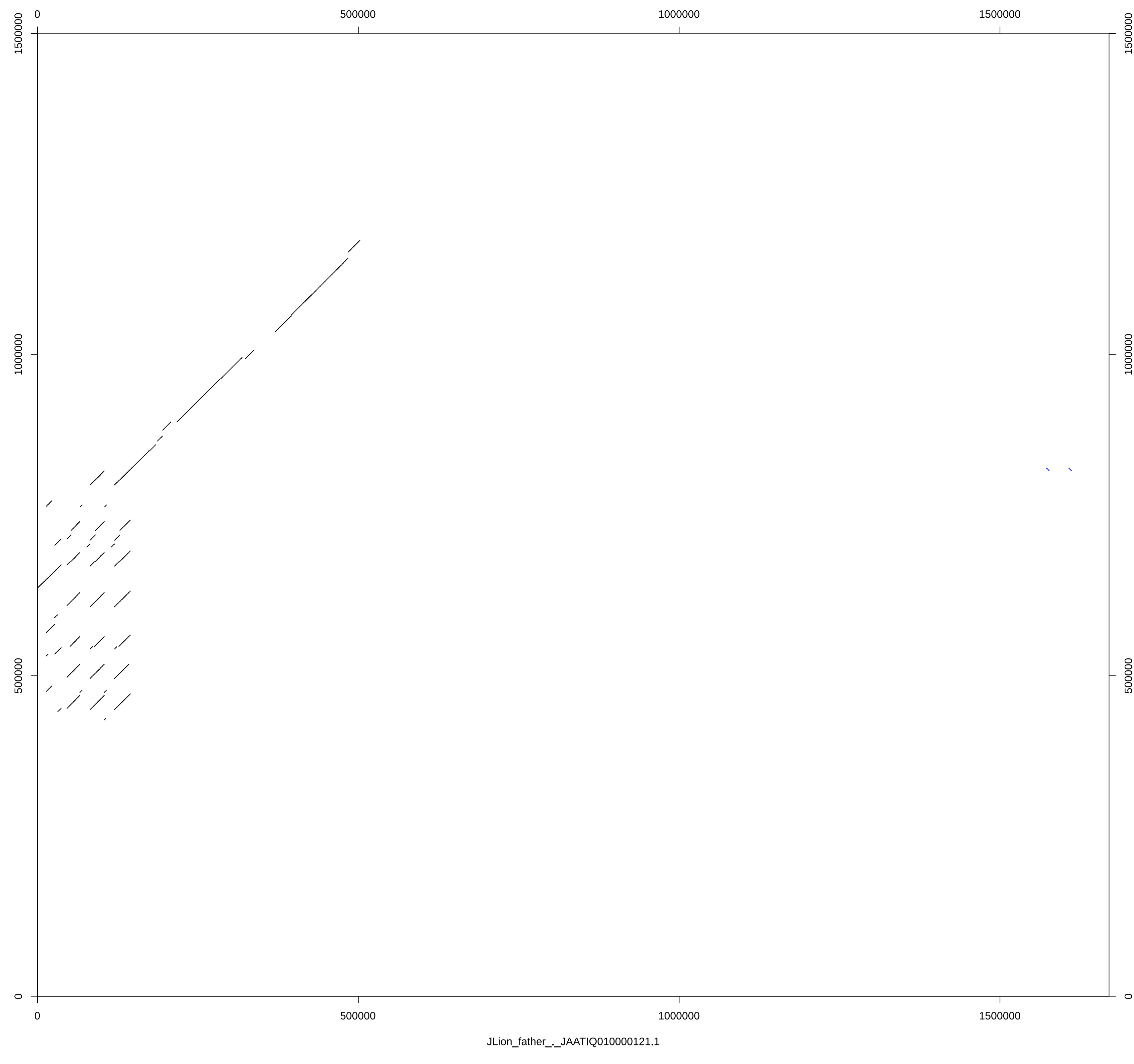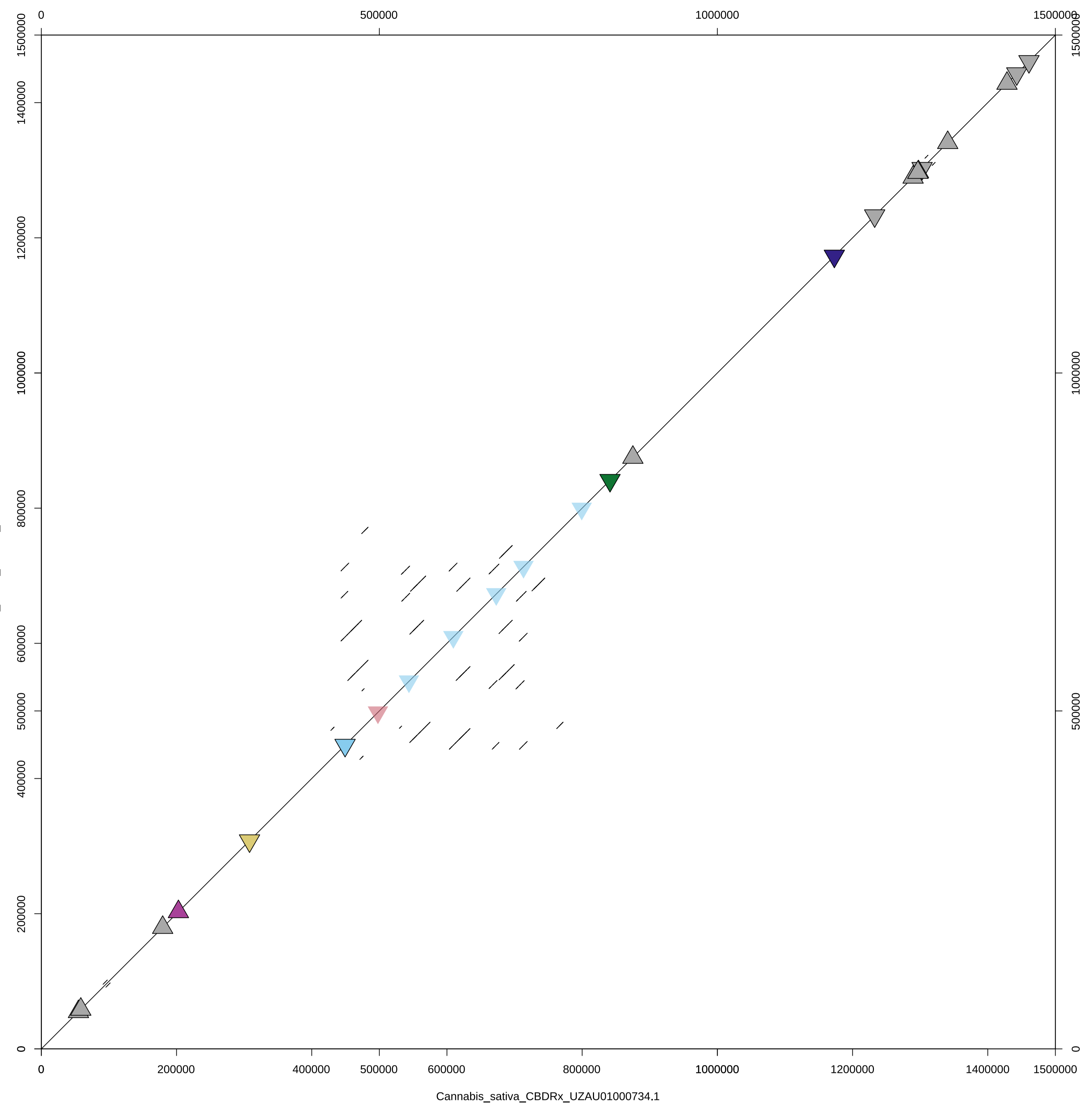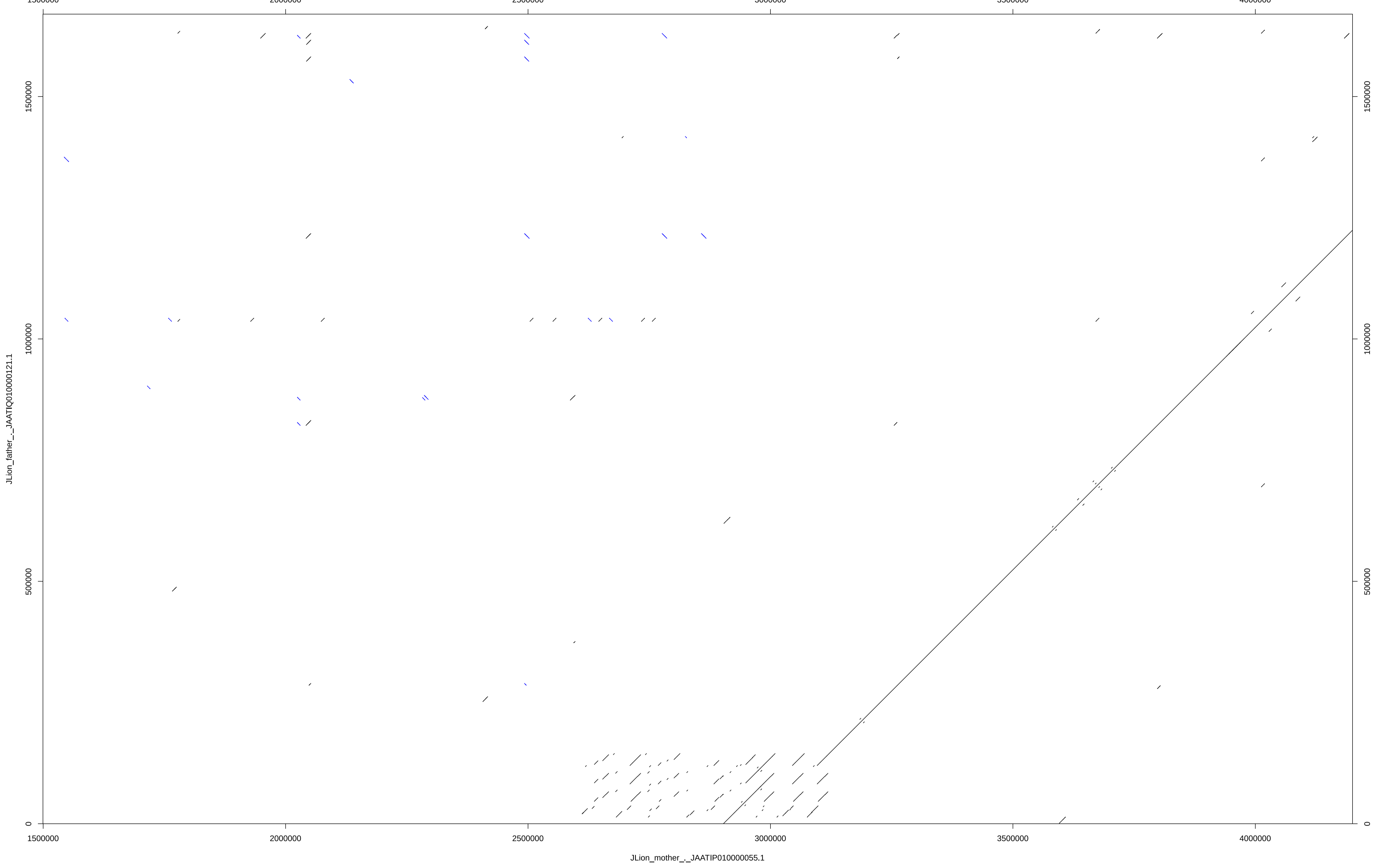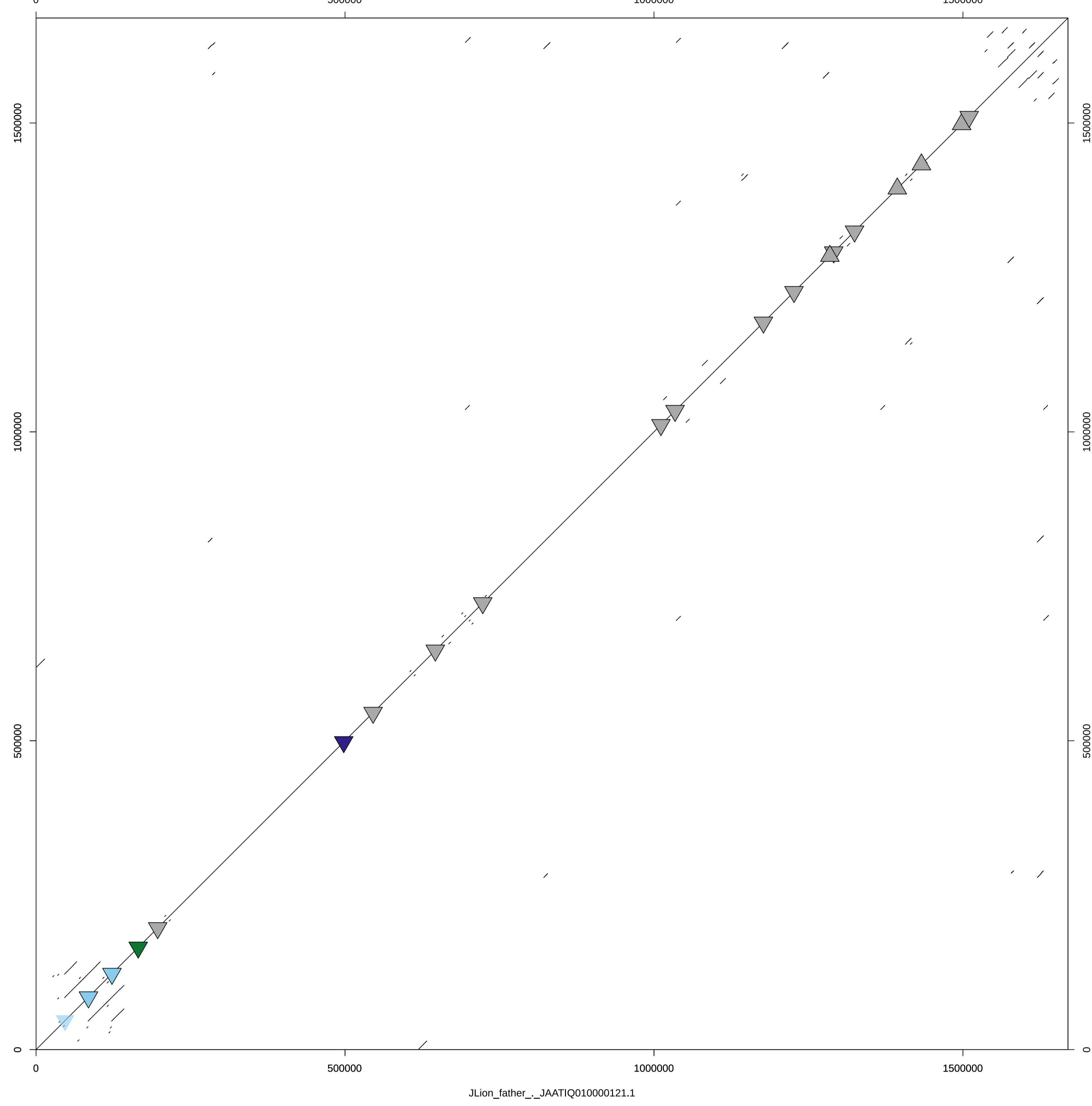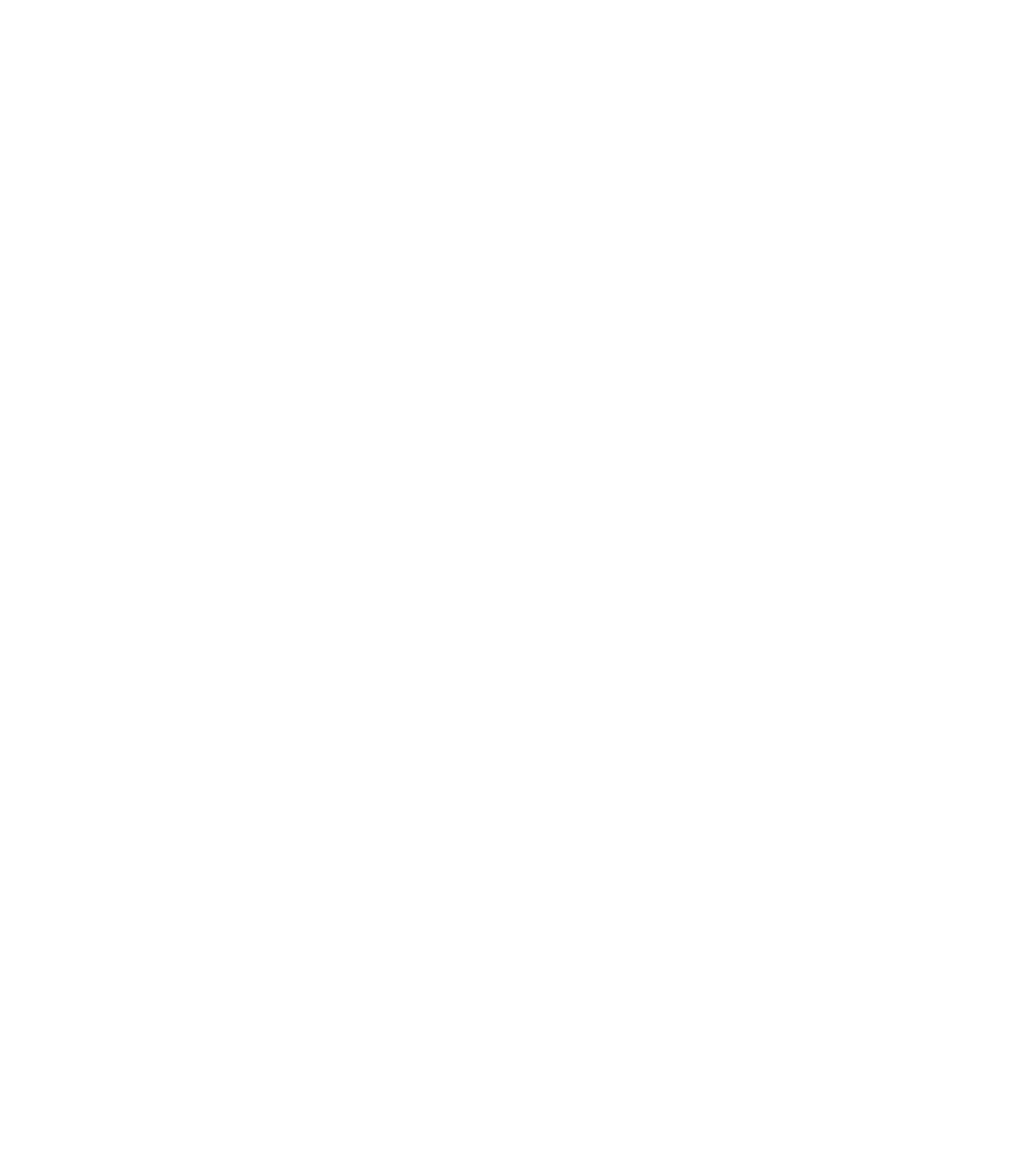

Jamaican Lion (mother)

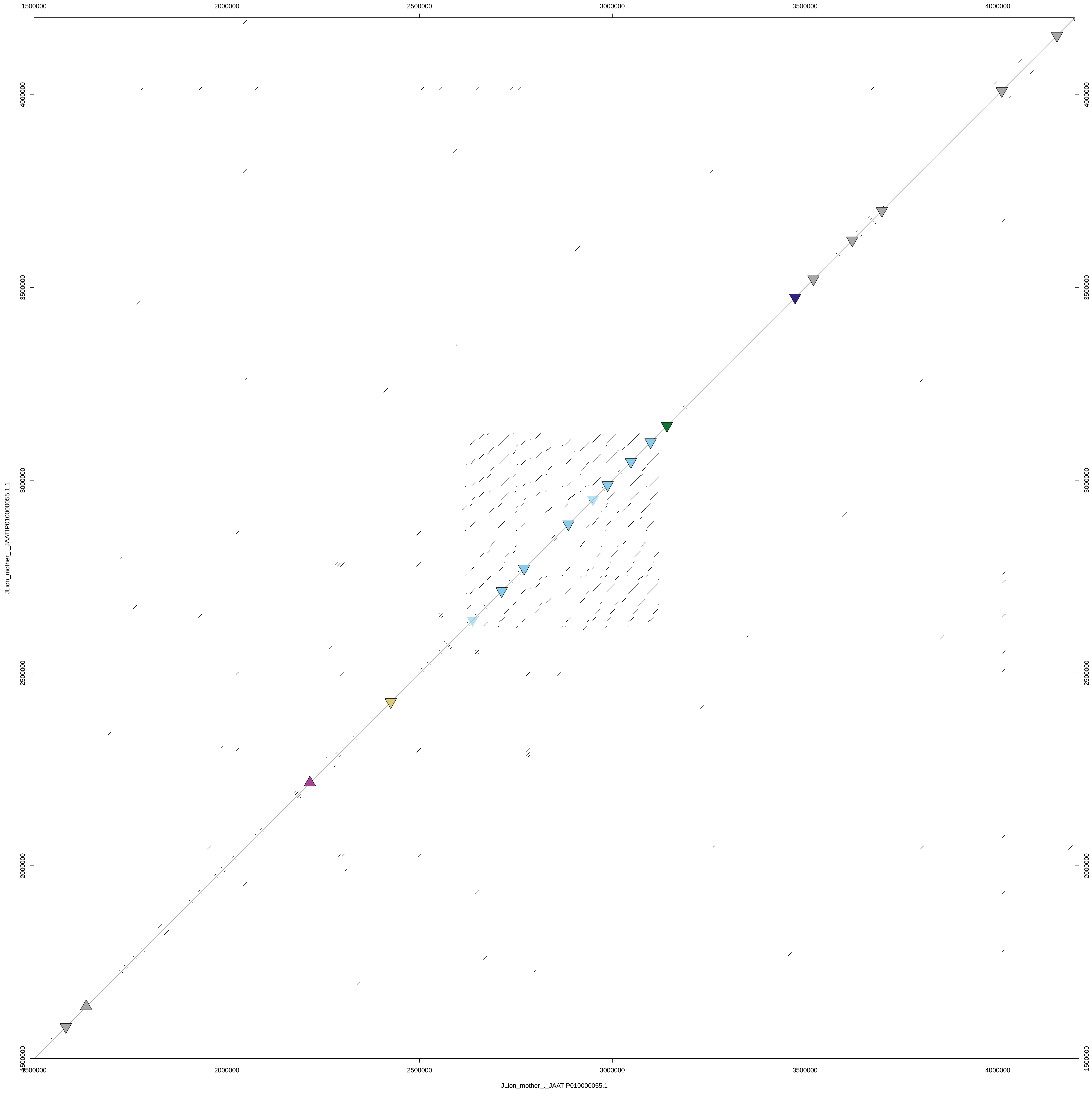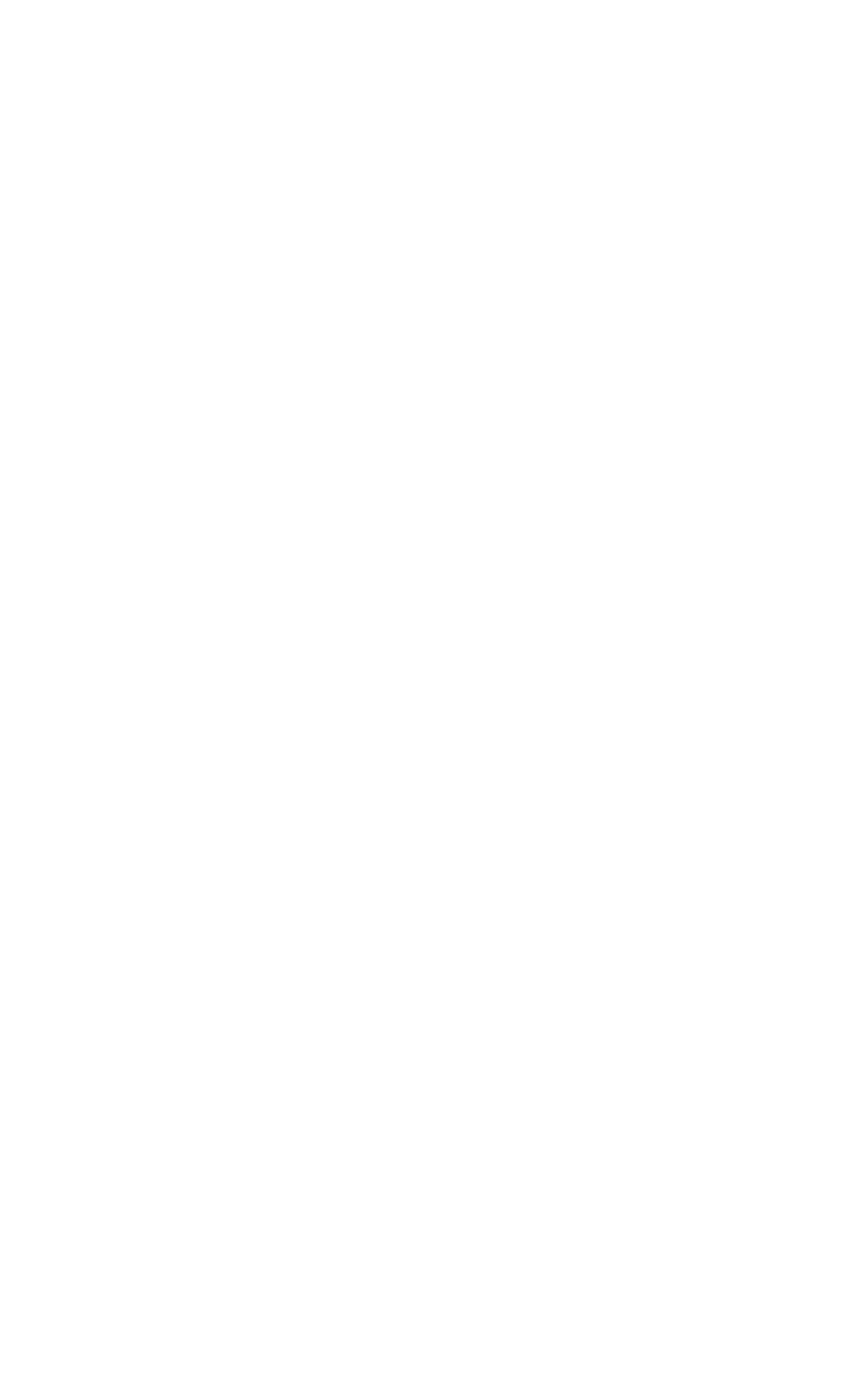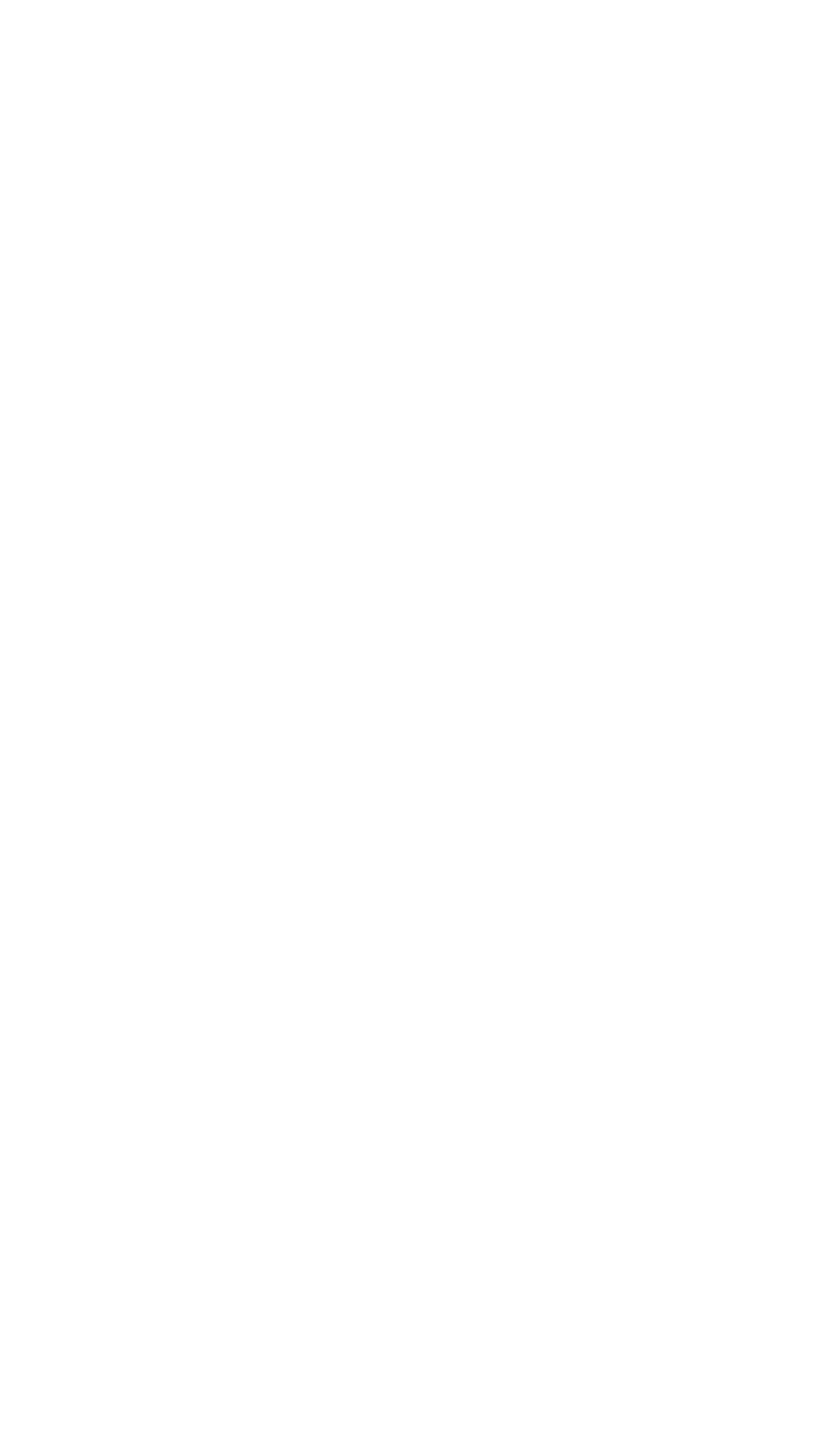

Supplement: evab130_Supplementary_Data [file evab130_supplementary_data.zip › Figure S4 - LASTZ nucleotide alignment dotplots of microsyntenic cluster 2.pdf]
