# Supplementary material for: Origin and Evolution of the Cannabinoid Oxidocyclase Gene Family
Source: Genome Biol Evol. 2021 Jun 8;13(8):evab130. doi: 10.1093/gbe/evab130 (PMC8521752; doi:10.1093/gbe/evab130)
Supplement: evab130_Supplementary_Data [file Figure S3 - LASTZ nucleotide alignment dotplots of microsyntenic cluster 1.pdf › Figure S3 - LASTZ nucleotide alignment dotplots of microsyntenic cluster 1.pdf]

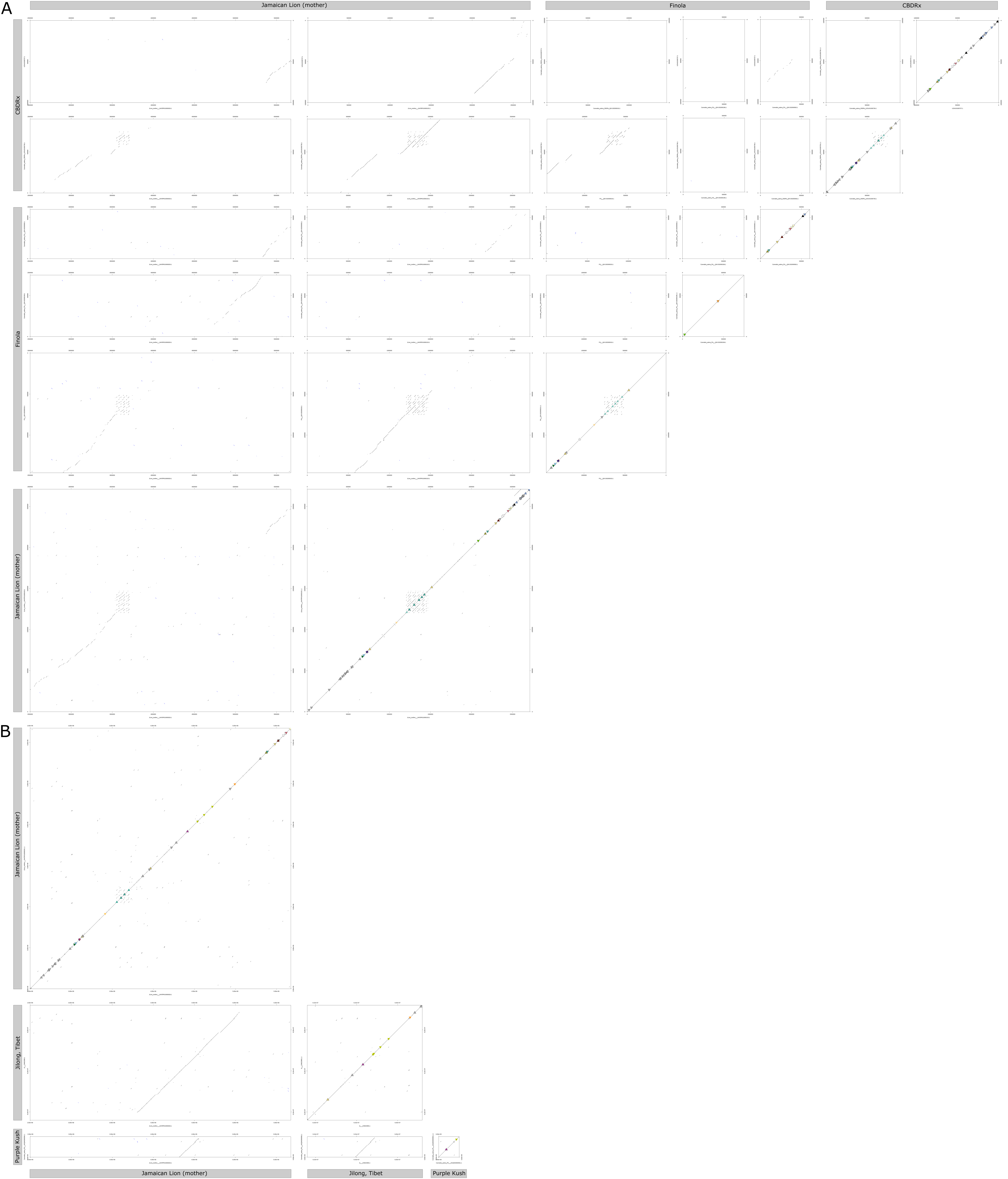
A
B
Jamaican Lion (mother)
Finola
CBDRx
CBDRx
Finola
Jamaican Lion (mother)
Jamaican Lion (mother)
Jilong, Tibet
Purple Kush
Jamaican Lion (mother)
Jilong, Tibet
Purple Kush
